# A New Method for Sensing Soil Water Content in Green Roofs Using Plant Microbial Fuel Cells

**DOI:** 10.3390/s18010071

**Published:** 2017-12-28

**Authors:** Natalia F. Tapia, Claudia Rojas, Carlos A. Bonilla, Ignacio T. Vargas

**Affiliations:** 1 Department of Hydraulic and Environmental Engineering, Pontificia Universidad Católica de Chile, Santiago 7820436, Chile; netapia@uc.cl (N.F.T.); cbonilla@ing.puc.cl (C.A.B.); 2 Centro de Desarrollo Urbano Sustentable (CEDEUS), Santiago 7520246, Chile; 3 Instituto de Ciencias Agronómicas, Universidad de O’Higgins, Rancagua 2840856, Chile; claudia.rojas@uoh.cl

**Keywords:** biosensor, green roof, microbial fuel cells, *Sedum*, soil water content

## Abstract

Green roofs have many benefits, but in countries with semiarid climates the amount of water needed for irrigation is a limiting factor for their maintenance. The use of drought-tolerant plants such as *Sedum* species, reduces the water requirements in the dry season, but, even so, in semiarid environments these can reach up to 60 L m^−2^ per day. Continuous substrate/soil water content monitoring would facilitate the efficient use of this critical resource. In this context, the use of plant microbial fuel cells (PMFCs) emerges as a suitable and more sustainable alternative for monitoring water content in green roofs in semiarid climates. In this study, bench and pilot-scale experiments using seven *Sedum* species showed a positive relationship between current generation and water content in the substrate. PMFC reactors with higher water content (around 27% vs. 17.5% *v*/*v*) showed larger power density (114.6 and 82.3 μW m^−2^ vs. 32.5 μW m^−2^). Moreover, a correlation coefficient of 0.95 (±0.01) between current density and water content was observed. The results of this research represent the first effort of using PMFCs as low-cost water content biosensors for green roofs.

## 1. Introduction

Over the last years, the expansion of urban areas has motivated the development of technologies such as green roofs, which in turn increases the amount of green areas in urban settlements and a series of other positive outcomes. Some of the additional benefits related to green roofs are runoff reduction [1], mitigation of heat island effects [2], sound and thermal insulation [3], creation of new habitats and increments in biodiversity [4], and an improvement in air quality [5]. These benefits explain the substantial expansion of green roofs in Western Europe, North America, and in some countries in the southern hemisphere, such as Australia and Chile [6,7].

In countries where climatic conditions are different to those of the northern hemisphere, mainly in rainfall and temperature, the construction and maintenance of these green areas should consider water availability during dry seasons. Even though the use of drought-tolerant plants, such as *Sedum* species, can reduce the amount of water needed for irrigation, there is still an excessive use of this resource in these systems. For example, a previous study, conducted in a semiarid climate during the dry season, demonstrated that an extensive green roof planted with *Sedum* could require up to 60 mm d^−1^ of water, which is approximately ten times larger than the evapotranspiration of reference [8]. For this reason, in semi-arid climates an efficient use of water is essential. To achieve this goal, continuous monitoring of water content in green roofs through water sensors provides a sustainable use of this critical resource. However, according to our experience monitoring green roofs [8,9], each of the typically-used sensors could cost from US$400 to US$1800; making this application less attractive for commercial and residential green roofs.

Microbial fuel cells (MFCs) are devices that allow bacteria to generate energy from organic and inorganic compounds. MFCs have been mainly studied for energy generation or power supply to sensors in remote locations, where battery replacement is not feasible [10]. In addition, these systems have been studied as an alternative for remediation [11], as biosensors of biological oxygen demand [12,13], chrome concentration [14], copper and cadmium concentration [15] and for corrosion monitoring [16]. 

Currently, these applications have been mainly tested under water-saturated conditions such as those proper of sediments, where water allows both the maintenance of anoxic conditions in the anode and the reduction of the internal resistance of the system [17], facilitating ionic mobility between electrodes. However, under unsaturated conditions, as is the case of many soil environments, MFCs have been less studied due to the high internal resistance as result of the poor conductivity the soil and the ionic mobility that these conditions impose. Thus, in these systems, power generation depends on water content, by decreasing the water content in soil, cell voltage and current decreases [18,19]. In spite of this, soil ecosystems have the potential to operate MFCs using plants in association with soil microbes (plant microbial fuel cells—PMFCs), taking advantage of the large soil biodiversity and the continuous supply of organic compounds by plant roots [20]. Therefore, considering the relationship between water content and current, a PMFC could be implemented to monitor the water content in constructed areas, such as green roofs, expanding the range of applications of MFCs.

Therefore, this study aims to evaluate the performance of PMFCs systems installed in a laboratory scale green roof under non-controlled environmental conditions. Performance was evaluated in terms of current generation and its relation with the water content of the soil and solar radiation.

## 2. Materials and Methods

### Plant Microbial Fuel Cell (PMFC) Construction and Operation

PMFCs were evaluated at a bench-laboratory and on a pilot scale in a green roof setting. The bench-laboratory experiments were performed prior to the pilot scale experiments under controlled temperature conditions (20 ± 2 °C) during 360 days. In these experiments, eight reactors consisting of plastic pots (450 mL) were filled with a plant growth substrate of a mineral matrix of 67% sand, 20% silt, 13% clay and 2.9% organic matter. Seven reactors had one seedling of each *Sedum* species (*S. kamschaticum*, *S. rupestre*, *S. album*, *S. hybridum*, *S. spurium*, *S. sexangulare* and *S. reflexum*) and one was left without plants as a control. These reactors were irrigated every week until the water drained from the bottom of the pot. Each PMFC consisted of two carbon-based electrodes: one buried at 7 cm made of circular fiber felt (92 mm diameter and 6 mm thickness); and a second electrode placed on at the surface of the substrate made of activated carbon granules (57 cm^3^ of volume) and graphite rod (6 mm diameter and 3 cm length) as connector. Titanium wires were used to connect the two electrodes to an external resistance of 1 kΩ. Reactors were inoculated with mud from a pond used as the water supply for irrigation located in the Campus San Joaquin at Pontificia Universidad Católica de Chile (PUC) in Santiago de Chile (33°29′ S, 70°36′ W) [9].

The pilot-scale experiments were conducted in the Laboratorio de Infraestructura Vegetal (LIVE), located in the Campus San Joaquín. LIVE consists of 17 modules (4 m^2^) mostly covered by *Sedum* species where the thickness (5–20 cm) substrate and four drainages systems commonly used in green roofs were tested [8]. To evaluate the relationship between soil water content and cell voltage, three identical modules with the same substrate depth (10 cm) and drainage system planted with the seven *Sedum* species previously described, were selected to set-up triplicate PMFC reactors (PMFC 1, PMFC 2 and PMFC 3) at 20–30 cm distance from the sprinkler used for irrigation. 

The reactors in LIVE were operated for 315 days. In the first 200 days, irrigation was applied automatically by the sprinklers, supplying 18 mm day^−1^, 60 mm day^−1^ and 15 mm day^−1^ between days 0–26; 27–136 and 137–200, respectively. After this time, sprinkler irrigation was suspended and water was supplied by natural rainfall.

Cell voltage was measured once per week during the first 280 days of operation using a multimeter UT55 (Uni-Trend Technology Limited, Dongguan, China). After that time, and for detailed measurements of the green roof PMFC reactors, data were collected every 10 min using data logger EM50 (Decagon^®^, Pullman, WA, USA). Power and current densities were normalized using the projected area of the buried carbon felt (circular electrode of 0.0067 m^2^). Polarization and power density curves were made, varying external resistance from 50 Ω to 80 kΩ every 45 min (chosen based on the period of time observed necessary to reach steady-state measurements in the bench-laboratory experiments). 

Environmental parameters such as solar radiation, temperature, relative humidity and precipitation were measured every 5 min by a photosynthetically active radiation (PAR) sensor, air temperature sensor (ECT) and high-resolution rain gauge (ECRN-100) (Decagon^®^, Pullman, WA, USA), respectively. Substrate water content, temperature and electrical conductivity were measured every 5 min by a GS3 sensor (Decagon^®^, Pullman, WA, USA). 

## 3. Results

The first section describes the relationship between water content and current density evaluated for the laboratory and green roof experiments. The following sections show the performance results for the experiments conducted on the green roof in relation to current generation and environmental parameters. 

### 3.1. Water Content and Current Density

**Figure 1:**
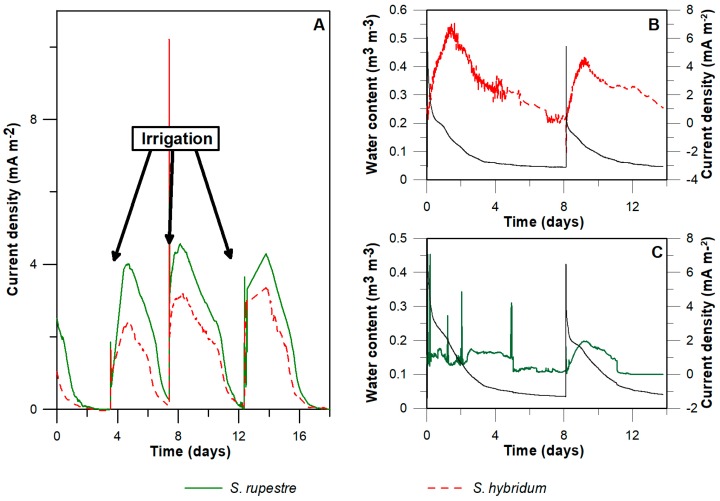
Relationship between current production and water content in laboratory plant microbial fuel cell (PMFC). (**A**) Current density of two *Sedum* species under controlled temperature conditions, in which cycles related with irrigation can be seen. On the right: Water content and current density during 14 days of *S. hybridum* (**B**) and *S. rupestre* (**C**). A decrease in the voltage with decreasing water content can be observed.

The current generated by PMFCs in the laboratory showed a tight relationship between current density and soil moisture content, which is reflected as an increment in the current as a result of irrigation (Figure 1A). Water content and current were continuously monitored for two PMFC reactors, representatives of the two higher-power densities produced by the PMFC reactors (i.e., *S. rupestre* and *S. hybridum*) [9]. Current peaks of 6.85 mA m^−2^ and 1.95 mA m^−2^ were observed after batch irrigation, reaching water contents of 16% *v*/*v* and 17% *v*/*v*, respectively. A current drop after about seven days was most clearly observed in *S. hybridm*, reaching zero when water content was less to 5% *v*/*v* (Figure 1B,C).

**Figure 2:**
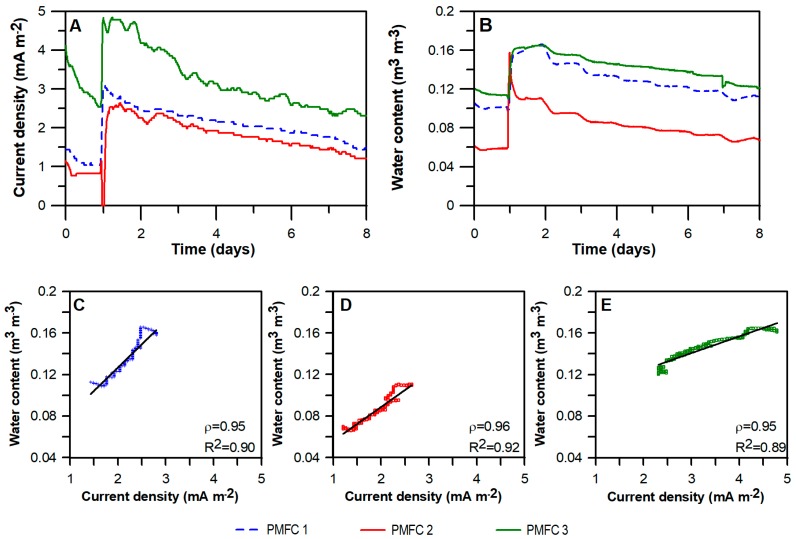
Correlation between water content and current density produced by PMFC reactors in Laboratorio de Infraestructura Vegetal (LIVE). In the upper part: Current density (**A**) and water content in the substrate (**B**) of three PMFC reactors located in LIVE during eight days. An increase in both variables due to rainfall is shown. Lower part: Analysis of correlation between current density and water content for the dates showed in the upper part of the three reactors: PMFC 1 (**C**), PMFC 2 (**D**) and PMFC 3 (**E**).

In contrast to the laboratory experiments, current batch cycles were not observed for the long-term experiments conducted on the green roof when irrigation was automatically supplied with sprinklers. This result is in agreement with our previous results for laboratory PMFCs operated under a continuous irrigation mode [9]. Interestingly, an increment in current density and water content as a result of rainfall was observed when the irrigation was suspended (Figure 2A,B). This observation enforces the idea of using PMFCs as water content biosensors for soils and substrates under unsaturated conditions, and especially for semiarid climates where low precipitation is expected.

During a period of eight days, current and water content were continuously recorded for the PMFC reactors located in the LIVE. By increasing the water content in the substrate, due to precipitation, an increase in the current density in all reactors was observed (Figure 2A,B), and a lower current was observed in the reactor with the lowest water content. Considering the current and water content values after the peak showed in Figure 2A,B, we carried out an analysis of correlation between both variables (Figure 2C–E). Correlation coefficient values of 0.95; 0.96 and 0.95 were obtained for PMFC 1, PMFC 2 and PMFC 3 respectively. Additionally, the R^2^ coefficient was calculated, obtaining values of 0.90, 0.92 and 0.89 for each reactor, demonstrating a linear relationship between considered variables.

### 3.2. PMFC Performance

**Figure 3:**
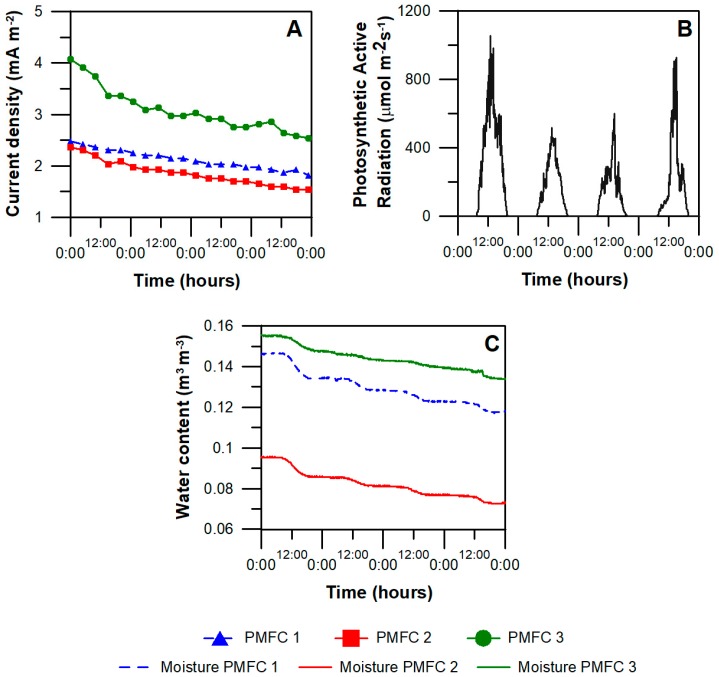
Performance of reactors located in LIVE between days 310 and 313 (**A**) together with the radiation (**B**) and water content in the substrate (**C**) in the same time period.

After one week of operation PMFCs 1, 2 and 3 showed a current density production of 0.06 mA m^−2^; 3.72 mA m^−2^; and 6.79 mA m^−2^; respectively. After, during day 100 to day 270, these reactors reached an average current density of 5.33 mA m^−2^, 4.11 mA m^−2^ and 6.73 mA m^−2^ respectively. Average photosynthetic active radiation observed during the testing period was 1423 ± 655 μmol m^−2^ s^−1^. Seasonal variations were observed, with an average radiation of 2106 ± 272 μmol m^−2^ s^−1^ during summer; 1103 ± 343 μmol m^−2^ s^−1^ in autumn; and 862 ± 346 μmol m^−2^ s^−1^ in winter. There was not a relationship observed between solar/light radiation and current produced by PMFCs (Figure 3A,B). However, the results suggest a slight increment in current overnight, similar to those observed in water content (Figure 3C).

### 3.3. Power Density and Polarization Curves

**Figure 4:**
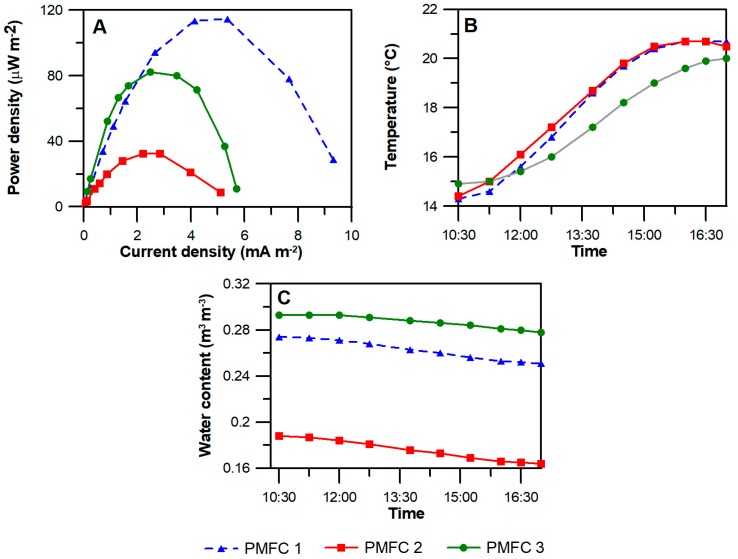
Power density curves (**A**) together with temperature (**B**) and water content (**C**) of the substrate during the six hours of the performance evaluation.

The power density curves (Figure 4A) showed maximum power densities of 114.6 μW m^−2^; 32.5 μW m^−2^; and 82.3 μW m^−2^ for PMFC 1, PMFC 2 and PMFC 3, respectively. The internal resistance of the system, obtained from the peak of the power density curve, was higher for PMFC 3, with a value of 2 kΩ, while in both, PMFC 1 and 2, was 0.6 kΩ. Similar substrate temperatures (Figure 4B) were observed in all reactors. A different water content was observed in the reactors (Figure 4C). While the lower value (17.5% *v*/*v*) was observed for the reactor with the lowest power density (PMFC 2), PMFCs with higher power densities had a higher moisture value (26.2% and 28.7% *v*/*v*, for PMFC 1 and PMFC 3 respectively). Although the water content allows the reduction of the internal resistance, providing better ion mobility, PMFC 3 showed the highest internal resistance of the replicates. This difference suggests that even though the three reactors were identically constructed and operated, slight differences in other factors, such as local substrate composition and the plant growth pattern could also affect soil conductivity or the electron transport to the buried electrode, consequently changing the value of internal resistance.

## 4. Discussion

The PMFCs performance showed differences in relation to previous studies in two aspects, that is, start-up time and radiation. The start-up period was shorter than those reported under laboratory conditions, where the start-up time was observed in more than 100 days [9,21]. This difference in the start-up may be due to the fact that the PMFCs tested in this study were installed on a green roof built about a year before the experiment, which could lead to a greater number of roots and therefore a higher organic matter content available for microorganisms, allowing the fast establishment of electroactive microorganisms (EAMs) in anodes. The solar radiation during the testing period was greater than that previously used in the laboratory for PMFC experiments, which does not exceed 596 ± 161 μmol m^−2^ s^−1^ [22,23]. However, a relationship between solar/light radiation and current was not observed in contrast to previous PMFC studies conducted with wetland plants [24,25]. There seems to be a slight increase in current during the night, which could be related to variations in the moisture content allowing an increase of ion mobility through the substrate or affecting the release of exudates from the roots. Previous studies had reported that the exudation of carbohydrates increases with the soil water content [26]. Crassulacean acid metabolism of *Sedum* might also lead to exudation of organic compounds in darkness periods when carbon is fixed. Hence, the plant metabolism can affect the exudation response to different factors, for example light intensity [27]. However, to the best of our knowledge, in the literature, there is no conclusive evidence related to this process. Further detailed research is needed to understand the root exudation of CAM species.

The maximum power density observed in PMFCs in LIVE (114.6 μW m^−2^) showed values lower than those reported for PMFCs using wetland plants (6–222 mW m^−2^) [28], that is expected considering no waterlogging condition. However, they are greater than those obtained under laboratory conditions using each of the seven species of *Sedum*, where *S. hybridum* achieved the highest power density (92 μW m^−2^) followed by *S. rupestre* (15.5 μW m^−2^) [9]. Similar substrate temperatures suggested that this parameter is not responsible for the observed differences in the power density curve, but there seems to be a relationship between two variables: power and water content, due to a lower value being observed for the reactor with the lowest power density (PMFC 2), while the PMFCs with the highest and the second highest power density had higher moisture values. However, PMFC 3, with the highest current density, did not have a significantly larger amount of water in the substrate, suggesting that there is an additional factor that could influence power generation. In laboratory experiments, differences in the anodic community were observed, where the PMFC with the highest power density showed a higher abundance of bacteria of the family *Micrococcaceae*, suggesting the importance of bacterial community structure for performance [9]. Dunaj et al. proposed that soil characteristics and bacterial community structure are key factors in the performance of soil-based MFC [29]. Further research is necessary to study the effect of these parameters on power production in PMFC systems settled in actual natural or engineered environments.

A similar behavior between current density and water content was seen, where an increase in the current and water content is observed as a result of irrigation. This is expected because the water allows better cation transport between electrodes and anoxic conditions in the anode [18]. Additionally, high correlation coefficient values were obtained, demonstrating a correlation between both the two variables. This suggests that PMFCs could be used as moisture biosensor, mainly in semiarid climate where water is a critical resource and efficient management is needed. 

Soil moisture is an important parameter because it not only affects plant growth and development, but also microbial activity and biogeochemical cycling of nitrogen and carbon [30]. For this reason, water content measurements are needed to keep a suitable water supply. The use of soil moisture sensors provides a reliable alternative for improving the water use efficiency, as water is applied in response to soil moisture variation instead of using a regular schedule. However, the commercial moisture sensors are relatively expensive for domestic or commercial applications in large natural and engineered environments. Thus, PMFC emerges as a new alternative to provide low-cost devices that could be designed covering large surface areas using granular carbon-based materials, without a negative impact on the soil/substrate microbiome [9]. Similar to conventional resistance type moisture sensors, which rely on variations in resistance to the current as a function of soil water content [31], the PMFC can measure the current generated, which changes in function of the soil resistance that relies on soil moisture. Although further studies and calibration steps are needed to design an optimized PMFC reactor, this type of biosensor could be a self-sustainable device, including a potential additional advantage in relation to conventional moisture sensors.

## 5. Conclusions

PMFC reactors operated under both controlled laboratory conditions in bench-size laboratory experiments and in a pilot scale green roof laboratory show that power generation is related to water content in the substrate under unsaturated conditions. Similar behavior between power generation and water content for two types of substrates were observed. Correlation coefficients of 0.95 (±0.01) were observed between current density and water content. Furthermore, reactors with higher moisture (26.2% and 28.7% *v*/*v* vs. 17.5% *v*/*v*) showed a larger maximum power density (114.6 and 82.3 μW m^−2^ vs. 32.5 μW m^−2^). Thus, the results of this research represent the first attempt at using bioelectrochemical technologies to develop a water-content biosensor, encouraging further research and innovation aimed to create affordable irrigation indicators and thus a more efficient use of water in green roofs or other urban green areas.

## Supplementary Materials

The supplementary materials are available online at http://www.mdpi.com/1424-8220/18/1/71/s1.
