# Supplementary material for: A New Method for Sensing Soil Water Content in Green Roofs Using Plant Microbial Fuel Cells
Source: Sensors (Basel). 2017 Dec 28;18(1):71. doi: 10.3390/s18010071 (PMC5795870; doi:10.3390/s18010071)
Supplement: Supplementary file 1 [file sensors-18-00071-s001.pdf]

A New Method for Sensoring Soil Water Content in Green Roofs Using Plant Microbial Fuel Cells

*Natalia F. Tapia[1,2], Claudia Rojas[1,2], Carlos A. Bonilla[1,2], Ignacio T. Vargas [1,2]**

SUPPLEMENTARY MATERIAL

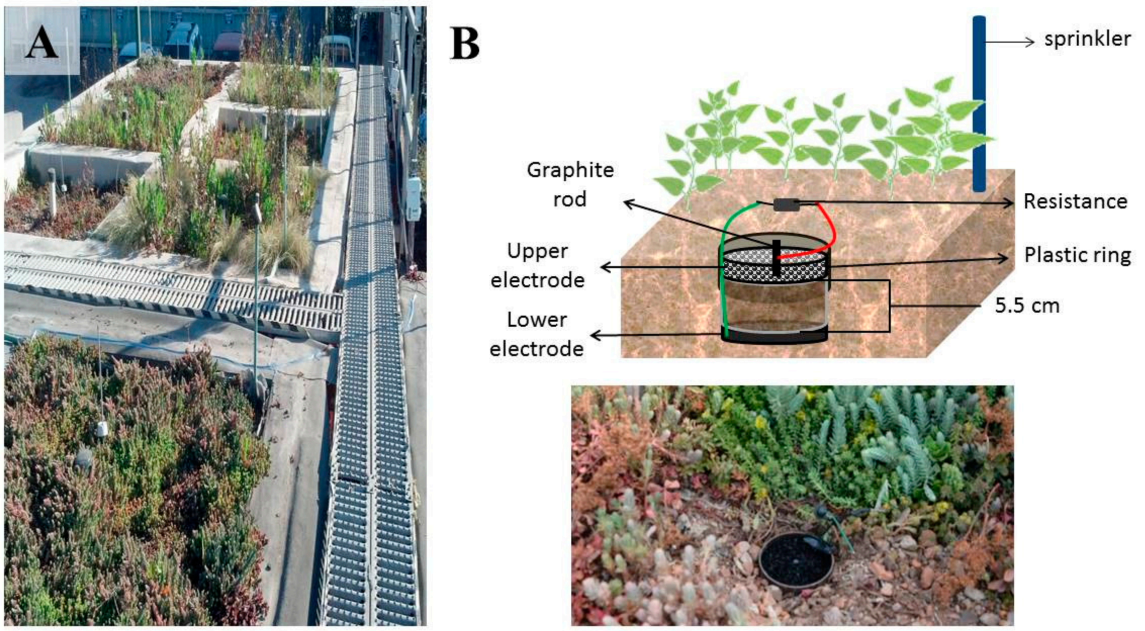

Figure 1S. Laboratorio de Infraestructura Vegetal (LIVE) located in the Campus San Joaquin at the Pontificia Universidad Católica of Chile (a). Diagram and photography of the PMFC reactor configuration installed in the LIVE (b).

Table 1S. pH, electric conductivity and anions concentration were measure in a solution 1:2 of three samples of the substrate of the Laboratorio de Infraestructura Vegetal (LIVE) finished the time operation of the PMFC reactors.

**Sample**	**pH**	**Electric conductivity ($mS\ cm^{-1}$)**	**Nitrate ($mg\ L^{-1}$)**	**Nitrite ($mg\ L^{-1}$)**	**Sulfate ($mg\ L^{-1}$)**
1	7.34	317	0.062	0.918	873.38
2	7.33	421	**	**	1599.39
3	7.19	465	0.974	**	1749.67
Mean±SD	7.29±0.08	401±76	0.35±0.55	0.31±.53	1407±48

** Concentrations under detection limit.

Note: Samples for anions analysis were filtered to 0.22 μm and analyzed by ion chromatography using an IC882 Compact Plus Chromatographer (Methrom, Switzerland).
